# The Role of RIN3 Gene in Alzheimer’s Disease Pathogenesis: a Comprehensive Review

**DOI:** 10.1007/s12035-023-03802-0

**Published:** 2023-11-23

**Authors:** Mostafa Meshref, Hazem S. Ghaith, Mohamed Ahmed Hammad, Mahmoud Mohamed Mohamed Shalaby, Faris Ayasra, Fatma Ahmed Monib, Mohamed S. Attia, Mahmoud A. Ebada, Hanaa Elsayed, Ali Shalash, Eshak I. Bahbah

**Affiliations:** 1 Department of Neurology, Faculty of Medicine, Al-Azhar University, Cairo, Egypt; 2 Faculty of Medicine, Al-Azhar University, Cairo, Egypt; 3 Medical Administration, University of Sadat City, Sadat City, Egypt; 4 Faculty of Medicine, Ain Shams University, Cairo, Egypt; 5 Faculty of Medicine, The Hashemite University, Zarqa, Jordan; 6 Faculty of Medicine, Assiut University, Assiut, Egypt; 7 Department of Pharmaceutics, Faculty of Pharmacy, Zagazig University, Zagazig, Egypt; 8 Faculty of Medicine, Zagazig University, Zagazig, Egypt; 9 Department of Neurology, Faculty of Medicine, Ain Shams University, Cairo, Egypt; 10 Faculty of Medicine, Al-Azhar University, Damietta, Egypt

**Keywords:** Alzheimer’s disease, Dementia, Ras and Rab Interactor 3, RIN3

## Abstract

Alzheimer’s disease (AD) is a globally prevalent form of dementia that impacts diverse populations and is characterized by progressive neurodegeneration and impairments in executive memory. Although the exact mechanisms underlying AD pathogenesis remain unclear, it is commonly accepted that the aggregation of misfolded proteins, such as amyloid plaques and neurofibrillary tau tangles, plays a critical role. Additionally, AD is a multifactorial condition influenced by various genetic factors and can manifest as either early-onset AD (EOAD) or late-onset AD (LOAD), each associated with specific gene variants. One gene of particular interest in both EOAD and LOAD is RIN3, a guanine nucleotide exchange factor. This gene plays a multifaceted role in AD pathogenesis. Firstly, upregulation of RIN3 can result in endosomal enlargement and dysfunction, thereby facilitating the accumulation of beta-amyloid (Aβ) peptides in the brain. Secondly, RIN3 has been shown to impact the PICLAM pathway, affecting transcytosis across the blood-brain barrier. Lastly, RIN3 has implications for immune-mediated responses, notably through its influence on the PTK2B gene. This review aims to provide a concise overview of AD and delve into the role of the RIN3 gene in its pathogenesis.

## Introduction

According to the latest updates from the World Health Organization (WHO), there are nearly 55 million dementia patients, with 10 million cases arising each year, and Alzheimer’s disease (AD) is the most prevalent type, affecting 60–70% of total dementia cases [1]. AD is a multifactorial chronic neurodegenerative disease associated with genetic and environmental factors, but most cases are sporadic [2]. Neuropathological specimens of AD patients showed atrophic changes caused by aggregation of misfolded proteins as amyloid plaques and neurofibrillary tau tangles (NFTs) [3], which were predominant in the hippocampus, frontotemporal cortical cells, the striatum, and the thalamus [4, 5]. AD patients often show cognitive disorders related to memory loss, inability to store new information, difficulty formulating thoughts and interpreting them into comprehensive speech, and problems with reading or paying attention [6]. They also develop behavioral changes like agitation, impulsive actions, and inappropriate language [7]. The symptoms can later worsen and involve physical disabilities such as urinary and fecal incontinence, infection, dysphagia, involuntary movements, and loss of communication [8].

The complexity of AD causation is increasingly understood, with over 20 genetic risk factors identified in addition to the previously known apolipoprotein E (APOE) [9–11]. Several newly discovered genes, such as Sortilin-related receptor 1 (SORL1), Protein tyrosine kinase 2 beta (PTK2B), Myocyte Enhancer Factor 2C (MEF2C), Bridging integrator1 (BIN1), Phosphatidylinositol Binding Clathrin Assembly Protein (PICALM), and Ras and Rab Interactor 3 (RIN3), are implicated in cellular processes like endocytic trafficking, underscoring the role of these pathways in AD pathogenesis [12]. For example, the amyloidogenic cleavage of amyloid precursor protein (APP) into toxic forms is regulated by endocytic mechanisms [13, 14]. Recent studies on RIN3, a guanidine nucleotide exchange factor, suggest its role in elevating the risk of AD, possibly by interacting with Rab5 to disrupt cellular trafficking and signaling [15–17]. Variants and expression levels of RIN3 have shown significant associations with AD, yet the exact mechanisms remain unclear. In this review, we aimed to summarize the current literature regarding the association between RIN3 expression and AD and possible mechanisms explaining this association.

## Brief Overview of Protective and Risk Factors of AD

### Risk Factors

**Fig. 1:**
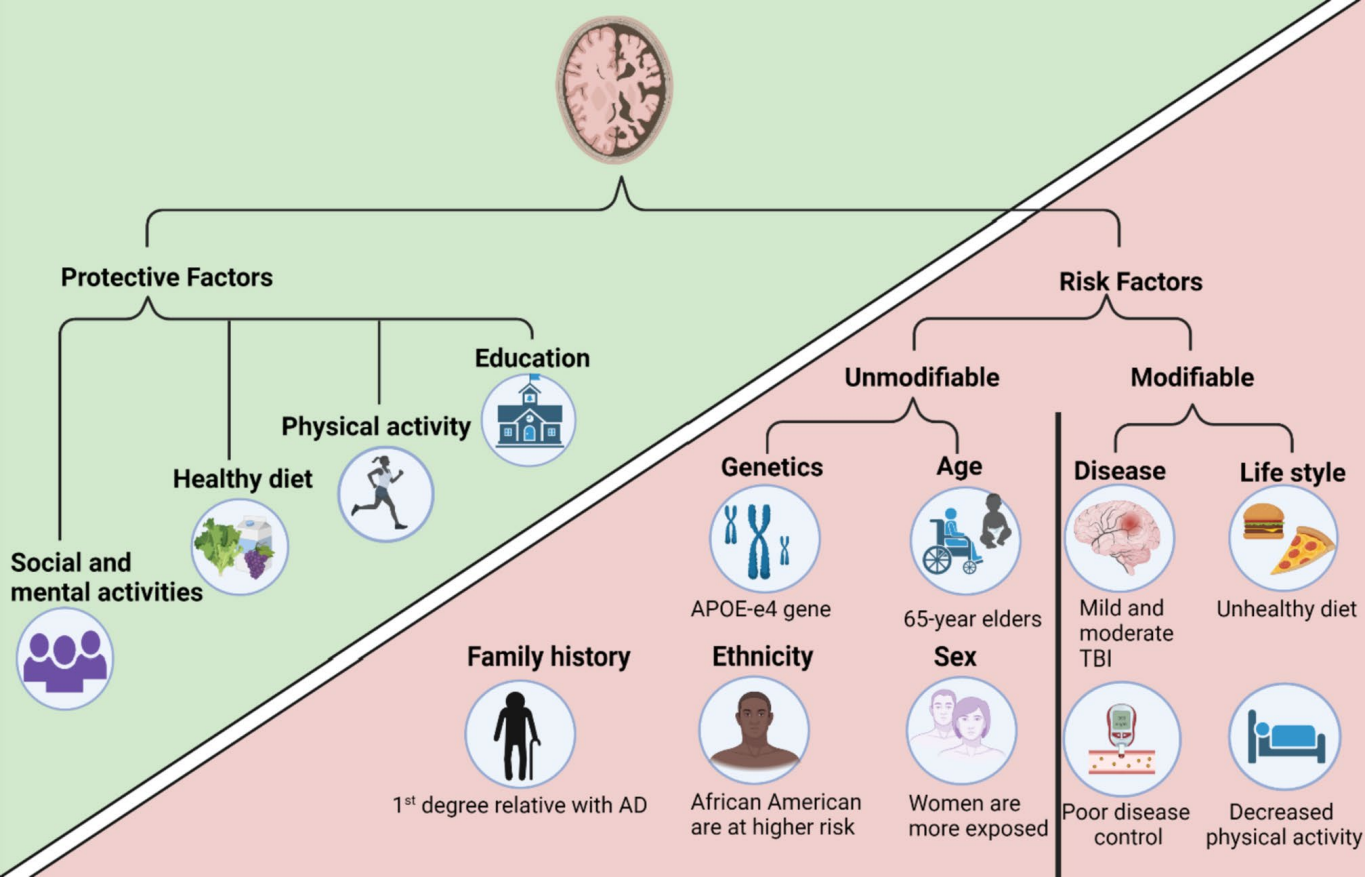
Protective and risk factors of AD

Several factors, both modifiable and unmodifiable, play a role in the onset and progression of AD (Fig. 1). The major contributors are age, genetic predispositions, and family history [18]. Specifically, individuals over 65 are at a heightened risk for developing AD, but age alone is not a conclusive factor and often interacts with other variables [19, 20]. Although the elderly are more prone to AD, the incidence rates can vary by country [21, 22]. Women in their eighties are more likely to have AD, and some studies suggest that this increased risk may also apply to women above 65 [23]. The genetic landscape is complex; while numerous theories seek to identify specific genes contributing to AD, the APOE-e4 allele remains the most substantial genetic risk factor for late-onset AD [18]. This allele is one of three forms of apolipoprotein inherited from each parent, and its expression may vary across ethnic groups, such as in African Americans [18].

A strong family history of AD, particularly in first-degree relatives, is another significant unmodifiable risk factor [24]. One large cohort study even indicated that having a family history could increase AD risk independently of the APOE-e4 allele [25]. However, the lifestyle practices within a family could also be a hidden contributing factor, thus complicating the significance of family history in AD development [18].

On the other side of the spectrum are modifiable risk factors, which are estimated to account for roughly 30% of all AD cases, although the direct links are not fully established [26]. These include lifestyle-related factors such as poor dietary habits, sedentary behavior, smoking, poorly managed diabetes, hypertension, and additional social and economic factors like inadequate education and poor mental health [18]. Traumatic Brain Injury (TBI) is another modifiable risk factor; patients with a history of TBI are twice as likely to develop dementia [27, 28], and mild TBIs can accelerate the onset of AD, especially when injuries are repeated [29, 30]. While the exact mechanisms remain unclear, chronic traumatic encephalopathy (CTE) resulting from repeated head injuries in activities like sports is suspected to affect cognitive and behavioral functions [31].

### Protective Factors

Physical exercise and dietary habits are often cited as protective factors against the onset and progression of dementia [32, 33]. Physical activity is thought to enhance blood and oxygen flow to the brain, although research has yet to specify the ideal type, frequency, or duration of exercise for maximal benefit [34]. The diet also plays a role in reducing the risk of AD; a heart-healthy diet that includes poultry, seafood, and whole grains while limiting unhealthy fats, sugar, and red meat is considered beneficial [35, 36]. Education is another protective factor, believed to establish a “cognitive reserve” that enables individuals to maintain higher levels of cognitive function despite brain pathology [37, 38]. Extended periods of education, particularly at a young age, coupled with other protective activities, have been shown to potentially lower dementia risk—even in the presence of high-risk genetic markers like the APOE-e4 gene [39]. Lastly, consistent social and mental engagement is recommended to foster cognitive reserve and enhance brain plasticity [40]. Multiple studies support the practice of daily social and mental activities as a way to fortify brain health and possibly delay the onset of dementia symptoms [33, 41].

#### Other Protective Factors

Several pathways exist by which APOE and APP mutations offer protection against AD. APOE is primarily produced by astrocytes in the central nervous system and carries cholesterol to neurons via APOE receptors, which are low-density lipoprotein receptors (LDLRs) [42]. The APOE protein plays a significant role in the metabolism of Aβ since it exerts a pronounced influence on the deposition of Aβ, leading to the formation of senile plaques and the development of cerebral amyloid angiopathy (CAA). These two pathological features are considered prominent indicators of amyloid pathology in the brains of individuals affected with AD [43].

The presence of the APOE ε4 allele is associated with a heightened susceptibility to developing AD [44]. The accumulation of Aβ in the form of senile plaques is found to be more prevalent in individuals who carry the APOE ε4 allele, as opposed to those who do not carry this allele [45]. However, the APOE 2 allele is still the strongest genetic protective factor against sporadic AD, and there are other APOE variants that offer protection as well [44]. In addition, it was observed that individuals who were homozygous for the APOE ε3 allele exhibited a protective effect against the development of AD. The traditional neuropathological manifestations associated with the APOE genotype include a greater accumulation of Aβ plaques and more pronounced cerebral amyloid angiopathy in individuals carrying the APOE ε4 allele. Conversely, those with the APOE ε2 allele exhibit a reduced burden of Aβ plaques compared to those who are APOE ε3 homozygotes [46].

Three decades of protection from AD have been demonstrated in a homozygous carrier of the APOE3 R136S variant (APOE3 Christchurch, APOEch), who also has the PSEN1 E280A mutation [47]. Emerging data suggests that certain less common variations of the APOE gene, namely APOE3 V236E (Jacksonville) and APOE4 R251G, may be associated with a reduced chance of developing AD. The APOE3-Jacksonville variant exhibits a propensity to decrease the self-aggregation of APOE, hence promoting its interaction with lipids and subsequently lowering amyloid burden and toxicity [48]. On the other hand, the presence of the APP protective mutation (A673T) has been potentially associated with enhanced cognitive functioning and reduced Aβ peptide pathology [49]. Recently, a PSEN1 E280A carrier with a heterozygous mutation H3447R in the Reelin gene (RELN) and a significant protective allele (RELN-COLBOS) was discovered [50]. No discernible mechanisms of protection in the RELN-COLBOS case were found to have altered the manifestation of pathology or the overall process of neurodegeneration. Nevertheless, it successfully retained the neural pathways essential for sustaining cognitive function well above the anticipated level for an individual in this particular group [50, 51].

Two remarkable examples of protection against AD dementia were possible because of the large size of the cohort and the disease heterogeneity in the PSEN1 E280A. Similar receptors (APOE Receptor 2 and VLDLR) and molecular pathways connect the two altered proteins, APOE and Reelin, and they may have similar mechanistic consequences, such as modulating tau phosphorylation via GSK3 [52]. Nevertheless, APOE is a significantly more prevalent molecule that could potentially elucidate the resistance to AD pathology. This resistance is hypothesized to be influenced by the widespread overexpression of APOE in astrocytes and microglia [53]. In contrast, Reelin primarily manifests inside distinct cerebral regions and cellular subgroups, engendering a confined safeguarding impact and promoting the viability of crucial neural networks that culminate in the postponed emergence of dementia, irrespective of the degree of AD pathology. This observation implies the existence of a resilient phenotype [51].

## AD Pathogenesis

The exact pathophysiology of AD is not well understood; however, the current popular hypothesis is the “amyloid cascade hypothesis,” which considers the accumulation of amyloid β-peptide (Aβ) plaques as the major player in AD pathogenesis [54]. The reported histopathological analysis of AD highlights two main characteristics of AD in the central nervous system: extracellular aggregates of Aβ plaque along with intracellular aggregates of hyperphosphorylated tau, forming neurofibrillary tangles (NFTs) [55].

The origin of Amyloid pathogenesis is the altered cleavage of APP by β-secretases (BACE1) and γ-secretases, producing the insoluble Aβ fibrils, which then aggregate, spread, and impair synaptic signaling [56, 57]. APP is a type 1 transmembrane protein with extracellular domains; its physiological function is related to neuronal cell survival and growth by the normal cleavage of APP [58, 59]. In the normal state, APP undergoes sequential cleavage by α-secretase, releasing a large soluble ectodomain called APPsα and C-terminal fragment C83 [59]. C83 cleaved further by γ- secretase forming soluble P3 peptide. Contrastingly, in the diseased state, APP is cleaved by β-secretase (BACE-1), releasing extracellular APPsβ and C-terminal fragment C99 [60, 61]. Further processing of C99 by γ-secretase yields the pathogenic Aβ; this APP processing pathway is called “the amyloidogenic pathway” [58, 59, 62].

Although the amyloidogenic pathway’s detailed regulation is not well explained, it is thought that excess production of Aβ induces aggregation of Aβ oligomers into polymers and eventually into insoluble plaques. Two main variants of Aβ peptides have a significant role in neurotoxicity: Aβ 40 and Aβ42; the latter is more likely to cause neuronal damage and plaque formation [63]. It is believed that Aβ plaques provoke secondary effects on the cellular level, such as oxidative stress, microglial activation, local inflammation, and hyperphosphorylation of tau protein, which further cause cell death and impairment of synaptic signaling [56, 57, 62]. It is believed that these secondary events may produce damage independently from their initial trigger (Aβ plaques) [64].

**Fig. 2:**
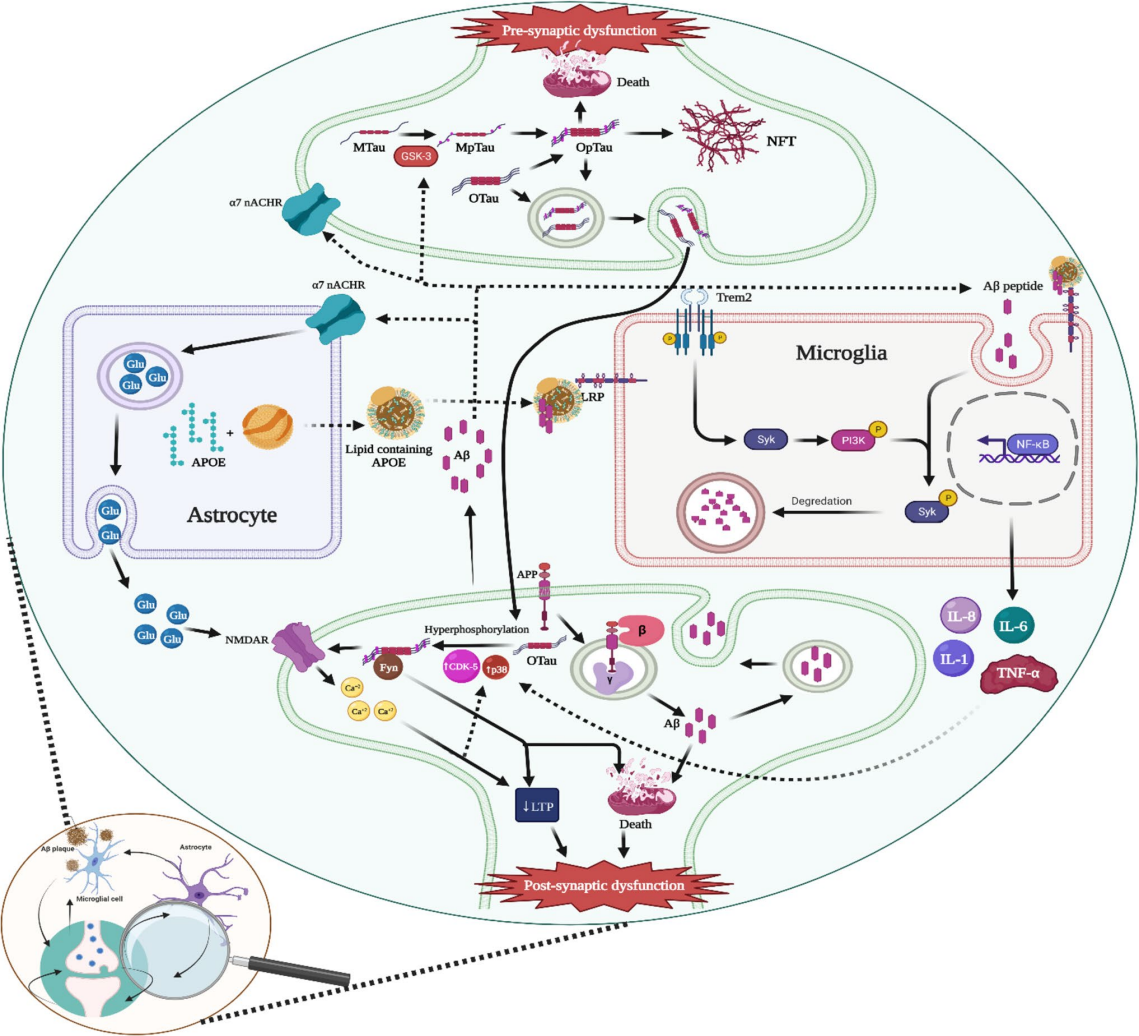
Molecular and cellular mechanisms underlying the pathogenesis of AD. The production of Aβ occurs through the enzymatic breakdown of APP within acidic cellular structures like late endosomes. Once released from neurons, this Aβ progressively forms more complex structures, beginning with oligomer aggregates (oAβ), advancing to fibrils, and finally culminating in the formation of amyloid plaques. Amyloid-beta oligomers (oAβ) impair synaptic function by weakening Long-Term Potentiation (LTP) and amplifying Long-Term Depression (LTD). Multiple neuronal receptors, including EphA4, PrPc, EphB2, NMDAR, and LiLRB2, have been identified as binding sites for Aβ, facilitating its toxic effects on synapses. Additionally, Fyn kinase serves as a key modulator for the neurotoxic effects mediated by NMDAR-bound oAβ. Furthermore, oAβ negatively impacts mitochondrial functions, triggering the activation of caspase-3, reducing ATP levels, and increasing Reactive Oxygen Species (ROS), which collectively exacerbate synaptic dysfunction. oAβ can stimulate microglia by binding to various potential receptors like TREM2, LRP1, RAGE, TLR4, and CD36. In particular, when Aβ interacts with TREM2, it activates the SYK signaling pathway via DAP12, a TREM2 adaptor protein, facilitating Aβ degradation. Microglial activation also results in the release of proinflammatory cytokines such as TNF-α, IL-1β, IL-6, and IL-8, which in turn can activate astrocytes. Additionally, oAβ may directly stimulate astrocytes via specific receptors like α7-nAchR, CaSR, CD36, CD47, and AQP4. Once activated, astrocytes could pose a threat to neurons through mechanisms such as altered extracellular glutamate homeostasis (also known as excitotoxicity) and the release of inflammatory molecules like TNF-α, IL-1β, and IL-6

One of the major pathologies in AD is impaired synaptic plasticity and loss of synapses, which is directly related to cognitive impairment — the main clinical manifestation in the early stage of AD [65, 66]. Notably, massive loss of synapses and postsynaptic receptors occurs early in AD, and it is assumed that postsynaptic receptors are crucial for Aβ effects on synapses [67–72]. AD is also defined by NFTs, which are straight, highly insoluble patches deposited in the dendrites of neurons and composed of tau protein. Normally, the Tau protein has a significant role in microtubule binding, synaptic signaling, and axonal transport [73]. Consequently, tau’s abnormal phosphorylation induces its polymerization and aggregation into NFTs, further disturbing signaling cascades, mitochondrial function, and neuronal communication [74, 75]. Fig. 2 summarizes the molecular and cellular pathogenesis of AD.

It should be noted that Aβ plaques control the phosphorylation of tau for NFT generation (Brien 2011). Pathological tau protein appears first in the basal forebrain, the brainstem raphe system, and locus coeruleus [76]. The progressive regional spread of NFTs is well described in the Braak staging system; NFTs are first detected in the transentorhinal cortex (Braak stage I) and entorhinal cortex (Braak stage II). Then NFTs progress to the hippocampus (Braak stage III), the middle temporal convolution, and the superior temporal gyrus (Braak stage IV). Finally, NFTs extend into the remaining cortex (Braak stages V and VI) [76].

## AD Genetic Background

AD is primarily categorized into two distinct forms: early-onset AD (EOAD) and late-onset AD (LOAD). The genetic characteristics of each differ substantially [77]. EOAD is predominantly caused by highly penetrant mutations in genes such as APP, presenilin 1 (PSEN1), and presenilin 2 (PSEN2) [78]. On the other hand, LOAD is more complex, involving multiple genetic risk factors. The apolipoprotein E ε4 (APOE ε4) allele is particularly notable for its significant association with the risk of developing LOAD [79]. Additionally, genome-wide association studies (GWAS) have identified various other genetic loci that contribute to the intricate genetic landscape of LOAD [79].

### Genetics of EOAD

The discovery of the correlation between LOAD development and genetic mutations in PSEN1, PSEN2, and APP provided important knowledge underlying the molecular mechanisms of AD pathogenesis.

#### APP

APP is located at the 21q21 chromosome and encodes a transmembrane protein type 1 [80]. APP enzymatic cleavage can result in Aβ formation that consists of 38 to 43 amino acids. APP sequential cleavage by γ- and α-secretase can lead to nonpathogenic peptide generation in a pathway called a constative or non-amyloidogenic pathway [81]. This pathway involves APP proteolysis by γ- and β-secretase, leading to sAPPα, Aβ, and c-terminal fragments formation. Dominant APP mutations represent about 14% of early-onset cases of AD, with about 35 mutations outlined that have been associated with the pathogenesis of AD [82]. These mutations involve duplications of the APP locus and various point mutations in the APP gene coding, which lead to the substitution of amino acids. Gene locus duplication can result in high levels of Aβ and APP and lead to an increase of Aβ1–42/Aβ1–40 ratio. Depending on the position of missense mutations, they can have variant effects [79]. If the amino acid is substituted by these mutations near the N-terminal of Aβ (the site of β-proteolytic cleavage), they result in increased production of Aβ, increased cleavage of β-secretase, and elevated aggregation and formation of the fibril [83]. If these mutations are close to Aβ’s C-terminal, the Aβ1–42 relative production will increase as compared to Aβ1–40. Also, in the Aβ domain, the Arctic mutation (E693G) occurs [84]. While the Arctic mutation fails to alter the Aβ42/Aβ40 ratio or elevate the Aβ levels, it likely elevates the mutant peptide aggregation rate [85]. All these APP mutations can provide further substantial evidence that the aggregation of Aβ is a crucial process in AD pathology. Furthermore, changes in the genetics that result in the APP alternation process and Aβ accumulation may lead to different neurovascular and neurological phenotypes [86].

#### PSEN-1 and PSEN-2

PSEN1 and PSEN2 are considered two homologous proteins located at 14q24.3 and 1q31-q42 chromosomes, respectively [87]. Also, they represent the crucial core of the γ-secretase complex, which has a critical role in the cleavage of APP into Aβ fragments. In addition to APP, they are also involved in some other protein cleavage, such as ErbB4, Notch-1, and proteins related to low-density lipoprotein receptors (LDLR) [88–90]. At the level of the cell, presenilins are localized in the Golgi apparatus, endoplasmic reticulum, and the nuclear membrane. It has been reported that mutations in presenilin genes are the most widely known cause of early-onset familial AD, particularly mutations in the PSEN1 gene, which accounts for about 80% of cases with early-onset AD [82]. Mutations in PSEN1 and PSEN2 proteins usually result in impairment in the activity of γ-secretase, which results in underproduction of Aβ1–40 and overproduction of Aβ1–42, and as a consequence of this, the Aβ42/Aβ40 ratio would be increased [91]. Mutations in PSEN1 protein have been correlated with the earlier ages of the disease onset (between 35 and 65 age), with an average of about 43 years [92].

### Genetics of LOAD

According to GWAS and multiple genetic studies, the majority of genes have been detected to be correlated with LOAD, such as clusterin (CLU), SORL1, CD33 antigen, APOE gene coding, and BIP1. However, there are other studies involving emerging genes, such as RIN3, whose function with AD is not completely understood and defined [93–96].

### Apolipoprotein E

One of the main risk genes for LOAD is the APOE ε4 protein, which is considered to be the CNS main apolipoprotein. This protein has a pivotal role in lipid transport and has a crucial role in the growth, maintenance, and reorganization of neurons. APOE ε4 allele carriers have an earlier age of AD onset. Moreover, they tend to have more marked amyloid plaque accumulation [97]. At the same time, the ε2 allele of APOE is correlated with a diminished risk of AD development, with decreased amyloid plaque accumulation [98, 99].

### CLU

CLU, a glycoprotein activated under stress conditions, engages in various physiological activities, including lipid transport, cell death regulation, inflammation, and membrane protection [100]. With respect to AD, CLU is believed to be implicated in its pathogenesis [101]. Studies indicate that CLU can form complexes with Aβ in the cerebrospinal fluid (CSF), which are capable of crossing the blood-brain barrier (BBB) [102]. GWAS has identified CLU as a potential biomarker for AD [9, 103]. Additionally, single nucleotide polymorphisms in the CLU gene have been suggested to influence AD pathology by affecting alternative splicing of the CLU gene [104].

### SORL1

SORL1, a member of the low-density lipoprotein receptor (LDLR) family, plays a critical role in the processing and trafficking of APP. Specifically, SORL1 is involved in directing Aβ toward lysosomal degradation [105]. Initially suggested as a potential biomarker for AD by Rogaeva et al. [106], this proposition has since gained validation through additional comprehensive research studies [95, 107].

### RIN3 Gene

RIN3, a protein-coding gene, is a member of the RIN family. The gene product is a newly identified binding protein that functions as Rab5 guanine nucleotide exchange factor (Rab5-GEF) [16, 108]. Rab5 is a member of the small GTPase Rab family that is localized usually to early endosomes [109, 110] and is implicated in homotypic fusion reaction of early endosomes and in clathrin-coated vesicle budding [111, 112]. RIN family is characterized by containing Ras-associating (RA) domain in their C termini, a region interacting with H-Ras [113, 114]. Also contains the SH2 domain in their N-terminal; thus, receptor-associated tyrosine kinases may adjust the functions of RINs through their interaction with tyrosine-phosphorylated receptors [115].

RIN3 has a sequence resembling RIN1 and RIN2. It includes SH2, proline-rich, RH, Vps9, and RA domains [115]. The same structure is preserved in RIN1 [116] and RIN2 [108]. RIN3 catalyzes a reaction of nucleotide exchange on Rab5 and prefers to react with the GTP-bound form of Rab5 [115]. The same interactions occur with RIN1 and RIN2 except for the special binding of RIN1 to the GDP-bound form of Rab5 [108, 117]. In addition to RIN2, RIN3 is considered to be an important factor for the stimulation and stabilization of Rab5 in the endocytic transport pathway [115]. Despite the wide expression of the RIN family, they are different in the distribution of their mRNAs, as RIN1, RIN2, and RIN3 mRNAs are widely distributed in the brain [116], heart, kidney, and lung [108], and in peripheral blood cells [115], respectively. RIN3 and RIN2 are restricted to endocytic vesicles with Rab5, especially when they are expressed in the cells, while RIN1 shows cytoplasmic distribution [115].

It has been found that RIN3 functions as a guanine nucleotide exchange not only for Rab5 but also for Rab31 [16]. It induces Rab31-bound GTPγS in the cell-free system and the formation of GTP-bound Rab31. In addition, RIN3 expression forms tubulovesicular structures that contain Rab31 in intact cells [16]. Additionally, RIN3 shows interaction with amphiphysin II as the N terminus of RIN3 that contains a proline-rich domain (PRD) linked directly with the SH3 domain of amphiphysin II. Such an important link is attributed to the class-II PRD included in both RIN3 and RIN2 but not in RIN1 [115]. Amphiphysin II interacts with amphiphysin I and forms heterodimers with it [118], and they are included in endocytosis, especially in synaptic vesicle recycling [119].

A number of novel loci, including RIN3, have been found to be involved in endocytic trafficking and signaling [17]. A meta-analysis of GWAS revealed that 14 genomic loci have been associated with AD. Nine of them have been formerly reported by GWAS, and Five of them (HLA-DRB5–HLA-DRB1, PTK2B, SORL1, SLC24A4-RIN3 and DSG2) were identified as novel loci. Findings showed that the BIN1 gene product, a protein involved in modulating tau pathology, interacts with the RIN3 gene and its encoding product [17]. Other GWAS studies marked the locus (rs10498633, G/T) upstream of the RIN3 coding sequences within its enhancer region. It is considered that such single nucleotide polymorphism (SNP) probably leads to increased expression of RIN3 in AD [17, 120].

## RIN3 in EOAD and LOAD

The cumulative evidence suggests the fundamental role of the RIN3-controlled endolysosomal pathway in AD [12, 17, 121]. A 2017 GWAS found a missense mutation in RIN3 (W63C) in sporadic EOAD [121]. Other genome-wide methylation studies indicated a group-wide hypo-methylation in RIN3, which was significant among the AD group compared to normal control [122]. Further, a recent experimental study proved the elevated expression of RIN3 in the hippocampus and cortex of AD animal models and even in the cultured cholinergic neurons of the basal forebrain. In addition, RIN3 was found to interact with both BIN1 and CD2-associated protein (CD2AP) to regulate APP trafficking and cleavage, which is associated with increased tau hyperphosphorylation. It is believed that RIN3 induces these effects by hyperactivation of Rab5 [17]. Taken together, little is known about the detailed pathogenesis of RIN in EOAD and LOAD; thus, further studies are required to improve our understanding of the complex nature of AD pathogenesis.

### RIN3 and AD Pathogenesis

Endocytosis is critical for the usual processes of APP formation and is considered an element of AD pathogenesis [123]. Significant numbers of multiple specific genes, including the RIN3 gene, have been identified. Such genes are encoded for endocytosis and their trafficking signals [12]. Initial discovery of the association of RIN3 with LOAD in GWAS in 2011 [10]. However, this association also can influence EOAD pathogenesis [17]. Nowadays, it is thought that RIN3 has contributed to AD pathogenesis (rather EOAD or LOAD) through different mechanisms that were discussed in multiple studies.

#### Up-Regulation of RIN3 Causes Endosomal Enlargement and Dysfunction in AD

RIN3 stimulates and stabilizes the Rab5 group-specific members (Rab5, 21, 22, 24, and 31) [16]. Rabex-5, which is (a GEF for Rab5) and Rabaptin-5 are needed for Rab5 activation. The overexpression of them is sufficient to cause early endosome enlargement [124]. Rab5 is a main organizer of endosomal incorporation and cellular transit [125, 126]. Excessive RIN3 expression enhances Tau phosphorylation. The increased RIN3 expression affects both the production of β-cleavage C-terminal fragment (βCTF) and the increase in pTau, and this is likely mediated by the Rab5 activation [17]. Early neuronal endosomal morphology seems to be important for controlling Aβ levels in neurons [127]. Normally, Rab5-marked early endosomes are the place where the processes of amyloidogenesis of the APP happen to produce Aβ, which occurs via the action of BACE1 and gives the βCTF. The late endosomes/trans-Golgi network (TGN) treats βCTF and produces Aβ, which is considered a toxic substance [13, 128]. The enlarged early endosomes showed a reactive immune response to different markers of early endosomes, including Rab4, Aβ, and EEA1. Thus resulting in the pilling up of Aβ in earlier stages of AD. Animal studies done by Grbovic et al. explained that early endosome enlargement could occur as a result of Rab5 overexpression, producing excess deposition of Aβ peptides in the cerebral blood vessels and brain tissue [128]. Studies of donated human tissues carried out via Cataldo et al. demonstrated that at the prodromal stages of AD, abundant neurons have a raised Aβ peptide deposition and show early Rab-5 positive endosomal enlargement [129, 130]. A recent animal module study by Shen et al. has explained that the upregulation of RIN3 and its increased expression in earlier periods of AD pathogenesis may result in Rab5 endosome enlargement (early endosome). Also, RIN3 interacted with BIN1/CD2AP to adjust the processing and trafficking of APP. Therefore, a considerable increase in RIN3 will affect APP endosomal trafficking and cleavage. This will induce degeneration of the neurons in earlier phases of AD [17].

The changes in the RIN3 gene can be due to DNA methylation, which has been associated with environmental stressors [131] and correlated with altered gene expression [132]. AS adding a methyl group to the gene at 3′ UTR has shown as a significant epigenetic sign that affects various genetic processes, including expression, transcriptional elongation, and splicing [133]. A proposal of irregular epigenetic framework participates in this procedure for many people. Poorly controlled different genetic functional processes can cause degeneration of nervous tissues. Thus, it is reasonable that odd variant genetic regulatory processes might trigger establishing disease pathology [134]. The AD brain enhances RIN3 manufacturing as a result of the co-existing amyloid medium by removing the methyl group from the 3′UTR genetic area. However, raised 3′UTRs methylation correlates to decreased RIN3 gene activity [133]. It was discussed before in a previous study done by Kirsty A. and his colleagues. They hypothesized and proved that RIN3 methylation is linked to abnormal gene function and increased risk and association in AD patients (mainly sporadic early onset AD), and there was relative hypomethylation noticed in the AD brain relative to blood [122]. In addition to that, multiple different genes of RIN3 were recognized in a previous survey, which was performed on 74,754 persons with BIN1 and CD2AP to be linked to the rising risk of variant causes of dementias, including AD [135]. The increasing evidence of RIN3 role has shown that RIN3 represents a significant part of AD pathogenesis. Genomic surveys had recognized a genetic site (rs10498633, G/T) upstream of the RIN3 coding sequences within its enhancer region. It is suspected that this single nucleotide polymorphism (SNP) is likely to result in overexpression of RIN3 in AD [120]. Also, a recent study showed a significant increase in the effect size of cognitively healthy centenarians compared to an age-matched group. The results were considerable for RIN3 (4.5-fold), but for APOE- ε2 were (2.2-fold) and for APOE-ε4 were (2.0-fold) [136].

#### RIN3 Impact on Transcytosis of Amyloid-β Through BBB β Transcytosis (PICALM Protein Pathway Affection) and Increase AD Risk

PICALM adjusts amyloid-BBB transcytosis and salvage via starting endocytosis, which is done by clathrin action through its interference with LRP1 (low-density lipoprotein receptor-related protein-1). LRP1 is a key amyloid-β clearance acceptor that also binds to APOE [137]. The PICALM pathway function in Alzheimer’s dementia confirms that clathrin-mediated endocytosis is a significant technique in amyloid-β salvage through the BBB [138]. This can be explained by the linking of amyloid-β to LRP1, promoting the PICALM binding. This started respectively PICALM/clathrin-dependent endocytosis of the amyloid-β-LRP1 combination and later transcytosis involving GTPases Rab5 and Rab11 controlling. Also leads to form early endosomes and exocytotic vesicles [139–141]. This BBB amyloid-β transcytosis required the integration of multiple genes, including PICALM, BIN1, CD2AP, and RIN3 genes [135]. Thus, it may be reasonable that biological effects that occur as a result of variant genetic disorders in the involved genes in the transcytosis processing pathway could increase AD risk because of amyloid-β aggregation in brain tissue [142–144]. Hence, increased RIN3 expression will affect the PICLAM pathway and lead to increased AD risk.

**Fig. 3:**
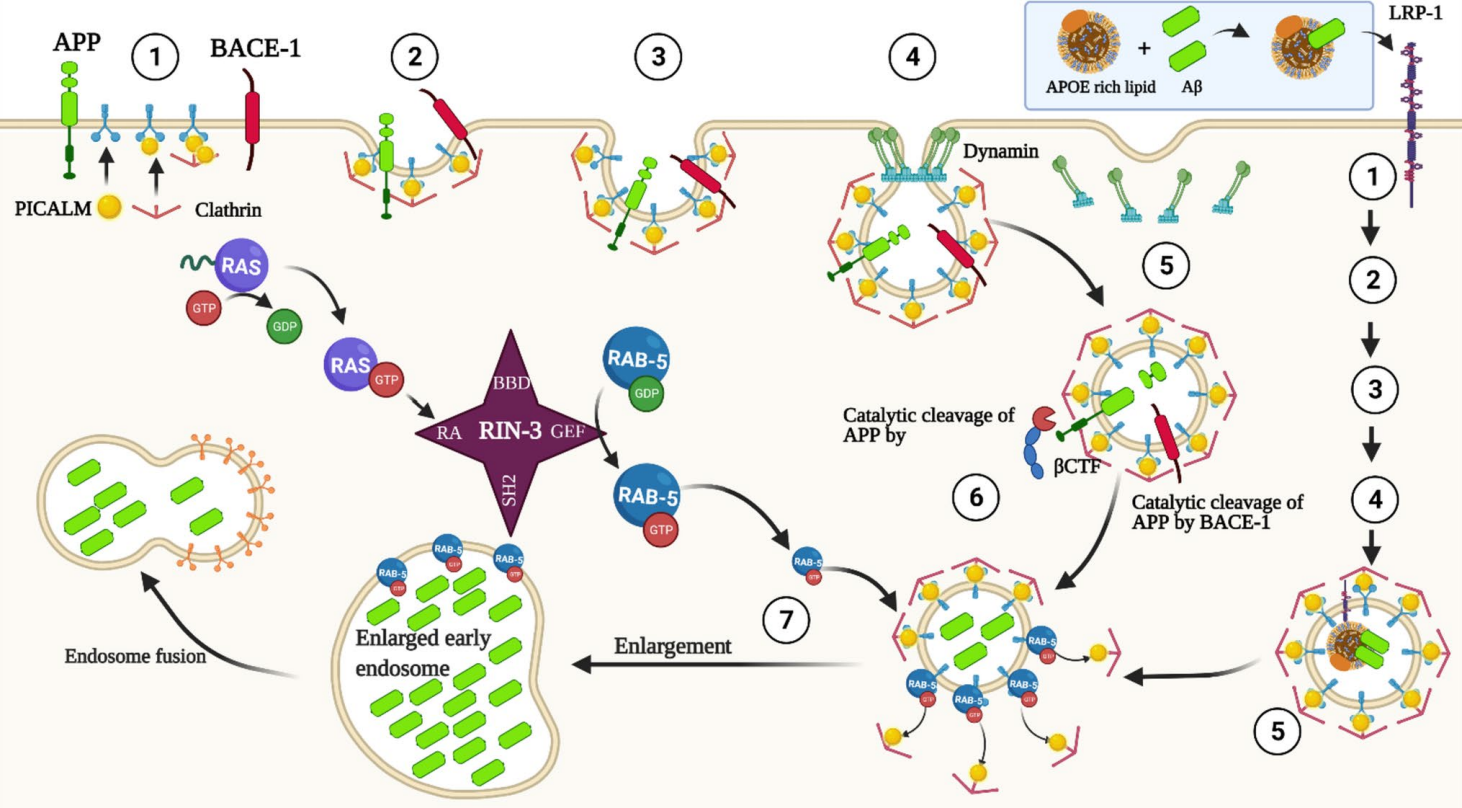
Molecular and cellular interaction between RIN3 and AD pathogenesis. When receptor tyrosine kinase (RTK) or tyrosine kinase (TK) is activated, it triggers the Ras/MAP kinase signaling pathway. Concurrently, RIN3's SH2 domain attaches to the phosphor-tyrosine residue (pY). This binding inhibits RIN3's GEF activity, keeping Rab5 in its GDP-bound state. On the other hand, when phosphotyrosine phosphatase (PPtase) deactivates RTK/TK, the Ras/MAP kinase signaling is dampened. Simultaneously, RIN3 detaches from RTK/TK, reactivating its GEF function. This promotes the transformation of Rab5 from a GDP-bound to a GTP-bound form. RIN3 collaborates with BIN1 and CD2AP to form a complex. Elevated RIN3 activity boosts Rab5 activation and subsequently triggers the assembly of the RIN3-BIN1-CD2AP complex on early endosomes. This leads to disruptions in endocytic trafficking. As a result, the RIN3-BIN1 complex activates GSK3β, contributing to tau phosphorylation. Concurrently, this disruption in trafficking promotes the cleavage of APP by BACE1 within early endosomes

Likewise, the PICLAM pathway is linked to regulating the function of the PTK2B gene (a cytoplasmic protein tyrosine kinase gene), which is an important gene contributing to AD risk through immune-mediated responses [145]. PTK2B plays a key role in the signaling cascade involved in the modulation of microglial and infiltrating macrophage cell activation [145, 146]. Therefore, RIN3 genetic affection will affect the PICLAM pathway and subsequently affect the PTK2B gene and increase the risk for AD. Figure 3 summarizes the cellular and molecular interaction of RIN3 with AD pathogenesis.

## RIN3 as a Biomarker for AD

Research findings have indicated that the expression of the RIN3 gene is significantly elevated in individuals with AD [17]. Furthermore, it has been observed that the abovementioned gene exhibits a state of hypomethylation within all peripheral blood samples obtained from individuals diagnosed with EOAD [122]. Hence, RIN3 could be a possible blood biomarker for detecting early AD.

The development of cognitive impairment was anticipated to be influenced by an abnormal expression of the RIN3 gene, according to a recent study. They explored the link between RIN3 gene methylation and early cognitive deficits post-transient ischemic attack (TIA) or mild ischemic stroke (MIS), assessing 84 patients within a week of the event using cognitive scales and comparing their RIN3 methylation status to 28 healthy individuals. Results indicated that TIA/MIS patients exhibited lower levels of RIN3 methylation compared to controls, with those experiencing early cognitive decline showing even more significant hypomethylation. These findings imply a potential predictive role for RIN3 methylation levels in identifying early cognitive impairment post-TIA/MIS, suggesting that modifying methylation through lifestyle or clinical means may alter the disease trajectory [147]. Thus, based on this data, the RIN3 could be a good indicator for early AD.

### RIN3 as a prognostic biomarker for AD

To improve patient treatment, genetic risk factors might be integrated into a diagnostic or predictive test for AD, allowing for more precise medical intervention (i.e., “genetic profiling”). This would not only provide light on the mechanisms involved in the etiology of the illness. The current feasibility of genetic risk profiling for AD diagnosis and prognosis is limited because the currently identified genes only explain a small proportion of the heritability of AD, and the level of discriminative accuracy considered acceptable is clearly dependent on the invasive nature of the treatment [82, 148]. Animal research has provided evidence to support the assertion that Aβ (amyloid-beta) plays a crucial role in both causing and being essential for neurodegeneration associated with AD [149, 150]. The regulation of APP trafficking and cleavage by RIN3 has been found to be linked to the occurrence of elevated tau hyperphosphorylation. The effects attributed to RIN3 are hypothesized to be a result of the hyperactivation of Rab5 [17]. Thus, RIN3 could be used as a probable prognostic agent for AD.

### Future Therapeutics Targeting RIN3

Several potential gene therapy targets exist for treating AD due to its complex genetic and environmental origins. These include the neurotrophic growth factors nerve growth factor and brain-derived neurotrophic factor; the amyloid beta-degrading enzymes neprilysin, endothelin-converting enzyme, and cathepsin B; and the AD-associated APOE [151]. Gene therapy for AD represents a valuable approach in the hope of an effective treatment that specifically addresses the fundamental causes of the disease. For several decades, the primary causative factors in AD and PD have been attributed to the presence of insoluble clumps of amyloid proteins [152]. In the same way, the majority of therapy strategies for AD are centered around the elimination of amyloid plaque [153].

The primary focus of the current research and development endeavors in the field of AD has been directed toward common approaches. (i) The main components implicated in the pathogenesis of AD include amyloid-beta and p-tau, as well as amyloidogenic proteases and other proteins that bind to amyloid-beta or tau, such as the receptor for advanced glycation endproducts (RAGE); (ii) The elements that are linked to pathology and are anticipated to play a role in the manifestation of symptoms include neuroinflammation, oxidative stress, and mitochondrial dysfunction; and (iii) Pharmaceutical interventions targeting cognitive and behavioral symptoms of AD encompass neuronal function modulators and neurotransmitters. However, it is important to note that these medications mostly provide palliative relief and do not directly address the underlying pathological reasons or offer a definitive cure [154].

Inhibitors targeting the b- and c-secretase enzymes involved in the processing of APP have progressed to phase II and III clinical trials; however, none have achieved regulatory approval as a pharmaceutical agent. Brain-permeable small molecule inhibitors of beta-site amyloid precursor protein cleaving enzyme 1 (BACE1), namely verubecestat, lanabecestat, and LY3202626, have demonstrated considerable efficacy in reducing the production of amyloid-beta (Ab) in individuals with healthy cognitive function as well as those with prodromal, mild, or moderate AD. However, these inhibitors were discontinued during phase II/III clinical trials due to their inability to effectively mitigate cognitive decline and the occurrence of adverse events, including weight loss, hair discoloration, psychiatric complications, and brain atrophy [155–157]. Given the important function of RIN3 in the generation of APP, it is reasonable to consider RIN3 as a promising candidate for future genetic therapy targeting AD.

## Post-Translational Modifications (PTMs) in RIN3 and AD Pathogenesis

Recent studies demonstrate the involvement of post-translational modifications (PTMs) in the pathogenesis and advancement of AD. Phosphorylation, glycation, acetylation, sumoylation, ubiquitination, methylation, nitration, and truncation are among the PTMs that have been shown in association with the pathogenic functions of proteins related to AD. Notably, these PTMs have been identified in relation to Aβ, BACE1, and tau protein, which are key players in the development and progression of AD [158, 159]. Disrupting PTMs, including phosphorylation, acetylation, glycosylation, and ubiquitination, will result in abnormal pathology during AD development and progression [160].

The expression of RIN3 is markedly increased and has a positive correlation with endosomal dysfunction in the APP/PS1 animal model. The modulation of RIN3 expression leads to modifications in axonal trafficking and processing of APP by means of its interaction with BIN1 and CD2AP [17]. Two separate research studies conducted on patients with AD have reached the conclusion that there exists a state of hypomethylation in the RIN3 gene, as well as three more genes. Furthermore, this research has postulated the potentiality that augmented manifestation of the wildtype RIN3 or manifestation of the RIN3 variation (W63C) could potentially play a role in the development of Alzheimer’s disease [122, 161]. In addition, it has been observed that upregulation of RIN3 leads to heightened activation of Rab5, which subsequently hampers the process of endocytic trafficking and signaling. Consequently, there is an elevation in the creation and storage of toxic APP-derived CTFs and an increase in the phosphorylation of tau protein. These processes collectively contribute to the degeneration of neurons in AD [17]. The alterations seen in the RIN3 gene may arise as a consequence of DNA methylation, a process that has been linked to the influence of environmental stressors [131] and correlated with altered gene expression [132].

## Can Coronavirus Disease 2019 (COVID-19) Play a Role in RIN3 Expression and AD Progression?

In an observational study, researchers tried to identify specific biomarkers associated with early-stage sepsis-induced acute respiratory distress syndrome (ARDS). They assessed the genetic profile and their expressed biomarkers in blood samples of severely ill patients on mechanical ventilators. They found about forty-one abnormally expressed genes specific to ARDS or sepsis, and RIN3 was one of the hub genes detected [162]. Several studies investigated the relationship between Severe acute respiratory syndrome coronavirus 2 (SARS-CoV-2) infection and sepsis, and many suggested a strong correlation between the virus pathogenesis and the clinical manifestations or biochemical changes of sepsis in critically ill patients [163, 164].

Since hypomethylation is the suggested mechanism behind the abnormal expression of the RIN3 gene in sepsis-induced ARDS [162] and SARS-CoV-2 is associated with Angiotensin-converting enzyme 2 (ACE2) hypomethylation [165], we may suggest a new genetic association between SARS-CoV-2 induced sepsis and AD. Our suggestion focuses on the severely infected population, but other studies are exploring the viral mechanism affecting the brain and the common mild inflammatory or immunogenic changes between viral pathogenesis and AD pathogenesis [166]. The proposed correlation involves retrograde transport of the virus from the olfactory bulb to areas with ACE2 receptors in the brain, such as the brainstem and capillary endothelium, damage to the BBB with inflammation [167], and exacerbating hypoxia injuring the hippocampus among other structures [166]. Anosmia in COVID-19 patients suggests a less severe course of the disease, but its presentation as a hallmark for both AD and COVID-19 is a warning sign of the potential risk of developing AD even in mild COVID-19 cases [168, 169].

## Challenges and Limitations in RIN3 and AD Research

However, research into developing medications or nonpharmacological treatments to prevent, stop, or slow down AD has remained unproductive despite breakthroughs in understanding the molecular basis of the illness. Both successful and unsuccessful clinical trials and rigorous pharmaceutical investigations are crucial because they either uncover prospective medications or exclude others, thereby pointing to the appropriate road to victory against AD [170, 171]. Factors that impede recruitment have been identified in studies on participation in Alzheimer’s disease research. Several factors contribute to the challenges faced in conducting research on Alzheimer’s disease. One such factor is the limited capacity and resources of primary care physicians to evaluate cognitive function and make appropriate referrals to research studies. Additionally, there are barriers that hinder the participation of under-represented communities, such as a lack of cultural sensitivity in the research process. Another obstacle is the necessity of having a study partner who can provide information on cognitive changes, which is a requirement for most Alzheimer’s trials. Furthermore, invasive procedures like lumbar punctures or brain imaging with injected tracer agents are utilized, further complicating the research process [172].

Primary care physicians are the first medical professionals most persons with cognitive impairment or memory issues will visit. According to studies, some of the challenges doctors face when referring patients for Alzheimer’s clinical trials are personal schedules, an absence of appropriate diagnostic clinical tools, worries about the safety of experimental protocols, patients’ multiple medical conditions, and their own geographical distance from a research facility [173].

The current attempts in drug development aimed at dealing with AD have encountered significant challenges in producing efficacious agents that modify the progression of the disease. These challenges arise from various factors, such as the considerable neuronal damage occurring prior to the onset of symptoms due to the buildup of the Aβ peptide and abnormalities in the tau protein. Additionally, adverse effects associated with drug candidates have proven to be detrimental, and the design of clinical trials has been insufficient in meeting the desired outcomes [174]. The anti-AD group should utilize nonpharmacological techniques based on modern technologies, such as noninvasive or less invasive surgical procedures. Neurogenesis is one mechanism by which a healthy lifestyle (including nutrition, sleep, and exercise) can delay the onset of AD [175].

## Future Directions

The current study establishes a foundational understanding of the role of RIN3 in AD, particularly in relation to Rab5 activation, tau phosphorylation, and amyloidogenic processing of APP. However, there are several gaps for future research. Although the study outlines the RIN3-BIN1-CD2AP complex formation, the precise molecular mechanisms through which RIN3 regulates Rab5 activity require further elucidation. Given the impact of RIN3 on endocytic trafficking, studies that explore the potential for therapeutic intervention targeting RIN3 or its associated complexes may prove valuable. Research involving patient cohorts is needed to validate the clinical relevance of RIN3 and its associated pathways in AD. Investigating how RIN3 interacts with other signaling pathways implicated in AD could provide a more holistic understanding of its role. Employing animal models could be beneficial to validate the biochemical changes observed and to assess the overall impact of modulating RIN3 activity.

## Conclusions

This review significantly advances our understanding of the role played by RIN3 in AD pathogenesis. We demonstrate that RIN3 not only activates Rab5 but also forms a complex with BIN1 and CD2AP, affecting both tau phosphorylation and APP processing. These findings underscore the potential of RIN3 as a key regulator in AD, opening new doors for future research and therapeutic interventions.

## Data Availability

All data are available within the manuscript.
